# Integrated Multi-Omics of the Longissimus Dorsal Muscle Transcriptomics and Metabolomics Reveals Intramuscular Fat Accumulation Mechanism with Diet Energy Differences in Yaks

**DOI:** 10.3390/biom15071025

**Published:** 2025-07-16

**Authors:** Jingying Deng, Pengjia Bao, Ning Li, Siyuan Kong, Tong Wang, Minghao Zhang, Qinran Yu, Xinyu Cao, Jianlei Jia, Ping Yan

**Affiliations:** 1 Lanzhou Institute of Husbandry and Pharmaceutical Sciences, Chinese Academy of Agricultural Sciences, Lanzhou 730050, China; ys230951330718@qhu.edu.cn (J.D.); baopengjia@caas.cn (P.B.); y220830390@xbmu.edu.cn (T.W.); 82101222366@caas.cn (M.Z.); ys230951330706@qhu.edu.cn (Q.Y.); 2 College of Agriculture and Animal Husbandry, Qinghai University, Xining 810016, China; ys240951330656@qhu.edu.cn; 3 Institute of Western Agriculture, Chinese Academy of Agricultural Sciences, Changji 831100, China; lining06@caas.cn; 4 Agricultural Genomics Institute at Shenzhen, Chinese Academy of Agricultural Sciences, Shenzhen 518000, China; kongsiyuan@caas.cn

**Keywords:** yak, longissimus dorsal muscle, intramuscular fat deposition, transcriptomics, lipid metabolomics

## Abstract

IMF (intramuscular fat, IMF), as a key index for evaluating meat quality traits (shear force and cooking loss, etc.), and its deposition process are jointly regulated by nutritional and genetic factors. In this study, we analyzed the molecular regulation mechanism of IMF deposition in the LD (longissimus dorsal muscle, LD) by dietary energy level in Pamir yaks. Meat quality assessment showed that the meat quality of the High-energy diet group (1.53 MJ/Kg, G) and the Medium-energy diet group (1.38 MJ/Kg, Z) were significantly improved compared with that of the Low-energy diet group (0.75 MJ/Kg, C), in which IMF content in the LD of yaks in G group was significantly higher (*p* < 0.05) compared with Z and C groups. Further analysis by combined transcriptomics and lipid metabolomics revealed that the differences in IMF deposition mainly originated from the metabolism of lipids, such as TG (triglycerides, TG), PS (phosphatidylserine, PS), and LPC (lysophosphatidylcholine, LPC), and were influenced by *SFRP4*, *FABP4*, *GADD45A*, *PDGFRA*, *RBP4*, and *DGAT2* genes, further confirming the importance of lipid–gene interactions in IMF deposition. This study reveals the energy-dependent epigenetic regulatory mechanism of IMF deposition in plateau ruminants, which provides molecular targets for optimizing yak nutritional strategies and quality meat production, while having important theoretical and practical value for the sustainable development of livestock husbandry on the Tibetan Plateau.

## 1. Introduction

Yaks live on plateaus above 3000 m above sea level, mainly distributed in the Qinghai Tibet Plateau of China [1]. China has a total of 16,556,000 yaks, accounting for more than 94% of the world’s yak resources [2]. Pamir yaks grow on the Pamir Plateau and are highly adaptable to high altitude, cold, and hypoxic environments, and they are characterized by good slaughtering performance and excellent lactation performance [3,4]. Yak meat is rich in protein and minerals with high nutritional value, and its consumption has been increasing in the past few years [5,6]. With the advancement of living standards, consumer demand for high quality meat has increased [7]. Therefore, enhancing the quality of yak meat has emerged as a prominent research focus.

IMF (intramuscular fat, IMF) deposition has been shown to exert a critical influence on improving the flavor and quality of meat. IMF is mainly distributed in the interstitial space of muscle fibers, and its composition is dominated by TG (triglycerides, TG) and PS (phosphatidylserine, PS) [8,9]. Research has demonstrated that IMF significantly influences key meat quality attributes, including flavor, juiciness, color, and tenderness. It is an important indicator of consumer concern [10]. As muscle fibers, adipose tissue, and connective tissue are cross-distributed in muscle, IMF deposition can relax connective tissue and reduce the physical hardness of muscle fiber bundles, thus reducing muscle shear and enhancing meat tenderness [11,12], while IMF-enriched phospholipids with long-chain polyunsaturated fatty acids can enhance meat flavor [13,14]. Therefore, IMF serves as a key factor influencing overall meat quality (edible quality and nutritional quality) parameters.

Dietary energy is a key nutritional limiting factor for animal growth and development [15], and its level has a significant effect on intramuscular fat deposition—the more energy livestock and poultry consume, the higher the fat deposition in their muscles [16]. It was found that increasing dietary energy levels significantly increased intramuscular fat content in yak beef and promoted intramuscular fat deposition by up-regulating intramuscular lipogenic gene expression and down-regulating lipolytic gene expression [17]. Feeding high-energy diets increased the intramuscular fat content of Holstein–Friesian bull beef and can be used as a way to produce high quality IMF beef [18]. Comparative analysis revealed significantly greater IMF content in beef from the High-energy diet cohort relative to the Low-energy group [19]. In summary, increasing feed energy levels significantly elevated muscle IMF content, resulting in improved muscle food quality.

Transcriptomics is a technique used to study all RNA transcripts of an organism [20], focusing on providing information such as the expression, structure, and function of genes to reveal the molecular mechanisms involved in specific biological processes [21], thereby enabling a deeper exploration of the biological functions of related genes [22]. Metabolomics is a technique for systematically identifying and quantifying all metabolites in a specific organism or organism sample [23], while lipid metabolomics is used to analyze the composition and expression changes of lipids in organism samples [24], while playing a role in exploring the lipid family and the functions of lipid molecules in biological processes [25]. At present, both transcriptomics and lipid metabolomics have been applied in domestic animals such as cattle [26], sheep [27], and pigs [28] and have achieved good results; Chen et al. [29] conducted lipidomics analysis on chicken preadipocytes treated with the *TMEM182* gene, revealing the metabolic effect of *TMEM182* on lipids in chicken preadipocytes. Zhao et al. [30] analyzed the longissimus dorsal muscle of pigs using transcriptomics and screened out potential candidate genes and pathways affecting the IMF. Zhang et al. [31] analyzed the longissimus dorsal muscle of Luchuan pigs and Duroc pigs using transcriptomics and lipidomics, emphasizing that the phenotype of intramuscular fat in Luchuan pigs being higher than that in Duroc pigs might be caused by key differentially expressed genes in the glyceride metabolism signaling pathway. These research results are of great significance for exploring the genetic functions of this species and improving its phenotypic traits.

In this study, 30 yaks from Ta County, Xinjiang were selected for group feeding, and meat quality trait indexes of the LD (longissimus dorsal muscle, LD) were determined by feeding different energy diets. In addition, based on transcriptome and lipid metabolomics analysis, DEGs (differentially expressed genes, DEGs) and SDLs (significant differential lipids, SDLs) were screened in the dorsal muscles of yaks in the groups Low-energy diet group (0.75 MJ/Kg, C), Medium-energy diet group (1.38 MJ/Kg, Z), and High-energy diet group (1.53 MJ/Kg, G) in order to elucidate the molecular mechanism of dietary energy-regulated deposition of IMF in yaks, and to provide theoretical support for the enhancement of meat quality.

## 2. Materials and Methods

### 2.1. Experimental Design and Sample Collection

In this experiment, 30 male yaks aged 3–4 years old in Ta County, Xinjiang were selected and randomly divided into three treatment groups—Low-energy diet group (0.75 MJ/Kg, C), Medium-energy diet group (1.38 MJ/Kg, Z), and High-energy diet group (1.53 MJ/Kg, G)—with 10 yaks in each group, and all the total mixed rations (TMRs) being formulated in strict accordance with the “Standard of Beef Cattle Breeding” (NY/T 815-2004) (see Table 1). The pre-test period was 15 days (concentrate was gradually increased) and the official period was 5 months. Three experimental yaks (a total of nine) were randomly selected from each treatment group and slaughtered after fasting for 24 h. The pH and color difference of the longissimus dorsal muscle of the yaks were measured for 45 min and 24 h. The LD was immediately divided into 1–3 cm^2^ tissue blocks and frozen in liquid nitrogen for storage, while samples of 2–3 kg were collected for meat analysis in the laboratory.

### 2.2. Longissimus Dorsal Muscle Quality Measurement and Hematoxylin–Eosin Staining (HE Staining)

The meat quality of the longissimus muscle tissue of the back of the yaks brought back was determined. The pH value was measured by inserting a portable pH meter 2 cm into the muscle and continuously for 3 to 5 times. Color determination was carried out using a TCP 2 fully automatic colorimeter. The color differences of meat samples at 45 min and 24 h were measured respectively. The results were expressed as L* (brightness), a* (redness), and b* (yellowness). To determine the cooking loss, the meat needs to be cooked in an 80 °C water bath to raise the internal temperature of yak meat to 75 °C for 45 min. Before cooking, the weight of the meat sample should be W1, and after cooking, the weight of the meat sample should be W2. The formula for calculating the cooking loss is as follows: cooking loss rate (%) = (W1 − W2)/W1 × 100%. The determination of shear force was carried out using the C-LM3B digital display muscle tenderness meter. The longissimus dorsi muscle was fixed with 4% paraformaldehyde and then stained with HE.

### 2.3. RNA-Seq Library Construction and Sequencing

A total of 9 tissue samples of the longissimus dorsal muscle (3 tissue samples in each group) were collected in this study and sent to Shanghai Ouyi Biomedical Technology Co., Ltd. (Shanghai, China). for transcriptome sequencing. The construction steps of the RNA-seq library are as follows:

After extracting the total RNA of the sample and digesting the DNA with DNase, the mRNA was enriched with magnetic beads of oligo (dT). After fragment synthesis, double-stranded cDNA was synthesized with six-base random primers. The library construction was completed after end repair, addition of A tail, and connection of sequencing adapters. After passing the quality inspection with Agilent 2100 Bioanalyzer, sequencing was performed using a sequencer to remove the adapter and low-quality reads. This was carried out as follows: Obtain clean readings, count the original sequencing volume and effective sequencing volume, and calculate the Q30 and GC content of clean reads. The clean reads were sequentially aligned with the specified reference genome using hisat2 2.1.0. The number of reads aligned to the protein-coding gene in each sample was obtained using htseq-count 0.11.2 software, and counts were obtained through alignment. The calculation of gene expression level was carried out using the FPKM method. Principal component analysis (PCA analysis) was conducted using the quantitative results of the genes. After obtaining the differentially expressed genes, GO functional significance and KEGG pathway significance analyses were conducted on them. The DESeq2 1.22.2 software was used to standardize the counts of each sample gene to calculate the multiple of difference, and NB (negative binomial distribution test) was adopted for the significance of difference test. Finally, the coding genes of differential proteins were screened based on the multiple of difference and the results of the significance of difference test. The conditions for screening differences are that the q-value is <0.05 and the multiple of differences is >2.

### 2.4. Lipid Extraction and Data Preprocessing

The process was conducted as follows: Take 30 mg of tissue, add 400 μL of pre-cooled methanol-aqueous solution, grind it, then add 400 μL of chloroform, vortex for 30 s, ultrasonically extract for 10 min, let it stand for 20 min, and then centrifuge for 10 min. Take 300 μL of the lower chloroform for volatilization and drying. Resolubilize the residue with 200 μL of isopropanol–methanol, vortex for 30 s, ultrasonicate in an ice bath for 3 min, stand at −20 °C for 2 h, centrifuge, and take 150 μL of the supernatant for LC–MS analysis. Metabolomics analysis was performed using a liquid chromatography–mass spectrometry system composed of ACQUITY UPLC I-Class plus ultra-performance liquid chromatography tandem high-resolution mass spectrometers. Chromatographic conditions were as follows: The chromatographic separation was performed at 55 °C using a binary solvent system—mobile phase A consisting of acetonitrile/water (60:40, *v*/*v*) with 10 mM ammonium acetate, and mobile phase B comprising isopropanol/acetonitrile (90:10, *v*/*v*) with 10 mM ammonium acetate. The MS data were collected using the Q Exactive LC-MS/MS system. The parameters of the MS system were as follows: Positive ion mode-Spray Voltage (V)—3500; Capillary Temperature—300 °C; Aux gas heater temperature—350 °C; Sheath Gas Flow Rate—45 arb; Aux gas flow rate—10 arb; S-lens RF level—50%; Mass range (*m*/*z*)—150–1500. Negative ion mode-Spray Voltage (V)—−3500; Capillary Temperature—300 °C; Aux gas heater temperature—350 °C; Sheath Gas Flow Rate—45 arb; Aux gas flow rate—10 arb; S-lens RF level—50%; Mass range (*m*/*z*)—150–1500. The data were preprocessed. Using the Lipid Search software V5.1, the raw format raw data exported by Q Exactive LC-MS/MS was read to obtain the precise mass numbers of MSn and parent ions. Based on the parent ions and multi-stage mass spectrometry data in each independent sample, the lipid molecular structure and the addition patterns of positive and negative ions therein were identified. The search results of each independent sample were aligned within a certain retention time range, and the results combined into a single report to organize the original data matrix. The missing value matrix is the data before the 0-value replacement and the log2 conversion, and the data matrix is the data after the 0-value replacement and the log2 conversion; this is used for subsequent analysis. After the data are normalized, then for the extracted data, where the ion peaks of each group of missing values (0 values) are greater than 50%, are deleted. The remaining missing values (0 values) are replaced with half of the minimum value, and the data processed with 0 values is subjected to log2 processing. Finally, the positive and negative ion data are combined into a data matrix table. This matrix contains all the information that can be used for analysis extracted from the original data, and subsequent analyses are based on this. The screening conditions of the data are *p*-value < 0.05, FC ≥ 1.2, or FC ≤ 1/1.2.

### 2.5. Real-Time Fluorescence Quantitative PCR (RT-qPCR)

A total of eight differentially expressed genes (DEGs), *COL11A2*, *HOXC10*, *FN1*, *FBN1*, *AGT*, *FOS*, *SCD*, and *SLC27A6*, were selected to verify the mRNA-Seq results by RT-qPCR. β-actin was used as the internal reference gene, and reverse transcription was performed using TransScript First-Strand cDNA Synthesis SuperMix (all-type gold) under the reaction conditions of 42 °C for 15 min, 85 °C for 5 s (1 cycle), and 4 °C for storage. Fluorescence quantitative PCR was performed using the LightCycler^®^96 SW 1.1 system, and the data were analyzed by 2^−∆∆Ct^ method. The primer sequences are shown in Table 2.

### 2.6. Statistical Analysis

One-way analysis of variance (ANOVA) was conducted on data such as the quality index of the longissimus dorsal muscle and the diameter of muscle fibers using SPSS 23 software. Multiple comparisons were selected. Pairwise comparisons were used among the three groups to reflect the level of difference, and the significance level was set as *p* < 0.05.

## 3. Results

### 3.1. Determination of pH, Color Difference, Shear, and Cooking Loss of the LD

Samples of three cattle were collected from each group. The meat quality of the longissimus dorsal muscle of Pamir yaks in groups C, Z, and G was measured. Each indicator of each cattle was measured 3 to 5 times. The data were screened and used for subsequent analysis. As shown in Table 3, the pH*_45min_ and pH*_24h_ values of the longissimus dorsal muscle tissue in groups G and Z of yaks were significantly lower than those in group C (*p* < 0.05), among which the pH*_45min_ and pH*_24h_ values in group G were significantly lower than those in group Z (*p* < 0.05). The color differences of yaks in group G and group Z (L*_45min_, L*_24h_, a*_45min,_ and b*_45min_) were significantly increased compared with group C (*p* < 0.05), among which the values of a*_45min_, a*_24h_, b*_45min_ and b*_24h_ in group G were significantly increased compared with those in group Z (*p* < 0.05). The shear force of the longissimus dorsal muscle tissue of yaks in group G was significantly lower than that in group Z and group C (*p* < 0.05). The intramuscular fat content in group G was significantly higher than that in group Z and group C, respectively (*p* < 0.05). These results indicate that an increase in the dietary level significantly improves the meat quality of the longissimus dorsal muscle of yaks, especially in terms of intramuscular fat content and tenderness.

### 3.2. Histology of the Longissimus Dorsal Muscles

**Figure 1:**
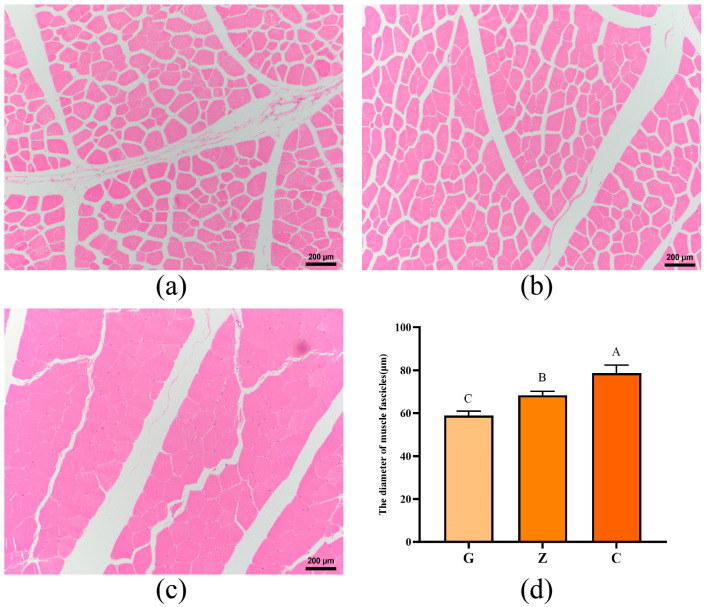
Histological analysis of the LD. hematoxylin–eosin staining of longitudinal sections of the longissimus dorsal muscle of yaks in groups G (**a**), Z (**b**), C (**c**). (**d**) Myofiber diameters of the LD of yaks in groups G, Z, C. Different letter marks indicate significant differences (*p* < 0.01).

Histological analysis showed that increasing the dietary energy level significantly increased the myofiber gap and decreased the myofiber diameter of the LD of yaks (*p* < 0.01). Measurement of muscle fiber diameter was carried out as follows: Measure the distances between the longest and shortest two points on the cross-section of each fiber, respectively. Randomly select three fibers and measure three sets of data for each fiber, and take the average value. Among them, the muscle fiber diameters of the High-energy (G), Medium-energy (Z) and Low-energy (C) groups were 58.87 ± 2.11, 68.36 ± 1.90, and 78.73 ± 3.74 μm, respectively (Figure 1).

### 3.3. Lipidomics Results and Analysis

**Figure 2:**
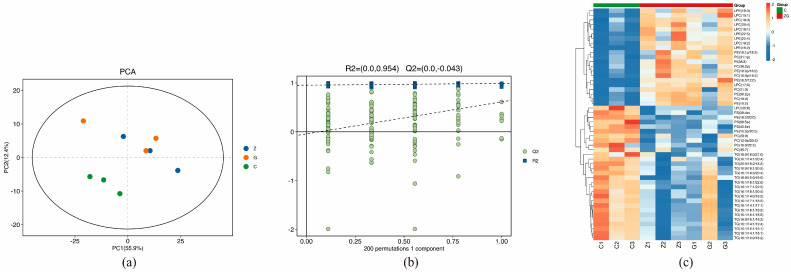
(**a**) PCA score plot showing lipid profiles in yak dorsal muscle across dietary energy groups (C: green, Z: blue, G: orange). (**b**) OPLS-DA permutation test results with overall sample scores; R2 (R-squared), Q2 (Q-squared). (**c**) Thermograms depicting lipid variations in yak muscle, where red and blue represent increased and decreased SDL concentrations, respectively.

The PCA results of the longissimus dorsal muscle and quality control (QC) samples from groups C, Z, and G are shown in Figure 2a. The OPLS-DA model was further constructed to prevent overfitting. As shown in Figure 2b, R2Y (cum) = (0, 0.954) and Q2 (cum) = (0, 0.43), which met the criteria for evaluating the validity of the model. All the green Q2 values on the left were lower than the original points on the right, indicating that the model was stable and no overfitting occurred; the above results showed that there was a significant difference in the longissimus dorsal muscle among the three groups, suggesting that the energy level of the diets significantly affected the lipids in the longissimus dorsal muscle of yaks. The lipid thermograms of the longissimus dorsal muscle among the groups C, Z, and G are shown in Figure 2c.

C, Z, and G were divided into ZvsC and GvsC for comparative analysis. A total of 86 differentially metabolized lipids were identified in ZvsC, among which 39 were up-regulated and 47 were down-regulated. KEGG pathway enrichment analysis was conducted on differentially metabolized lipids. The results showed that a total of 14 KEGG pathways were enriched, mainly distributed in metabolism, cellular processes, and organismal systems. Fifty-three differential metabolites were identified in GvsC, among which 25 were upregulated and 28 were downregulated. KEGG pathway enrichment analysis was performed on differential metabolic lipids, and the results showed that a total of six KEGG pathways were enriched, mainly distributed in metabolism and cellular processes.

The key lipids screened out by the two comparison groups included TG(17:0/18:1/22:5), TG(18:1/17:1/18:3), TG(16:1/18:1/22:6), TG(16:0/10:0/16:0), TG(16:0/14:0/17:0), and TG(20: 5/18:2/18:2), PS (39:4), PS (38:5e), PS (38:4e), PC (12:0p/20:5), PE (16:0p/20:5), PE (18:0/17:1), PE (16:0p/20:5), PE (33:1p), and PE (17: (1/18:0)).

### 3.4. Transcriptomics Results and Analysis

**Figure 3:**
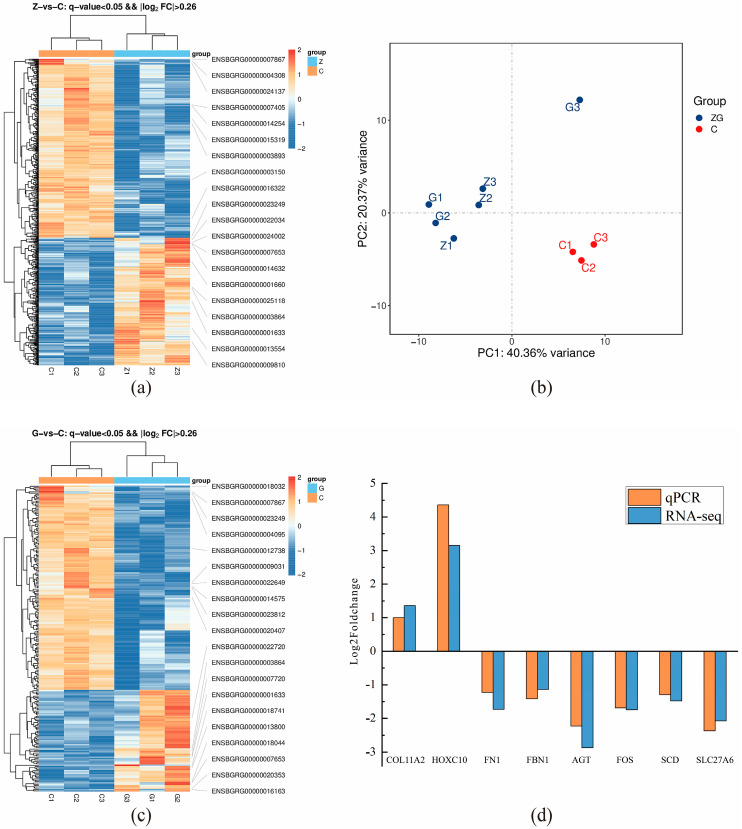
(**a**) Heatmap of differentially expressed genes in the longissimus dorsal muscle of yaks in groups C and Z, (**b**) PCA score plot of differentially expressed genes in yak dorsal muscle across dietary energy levels. (**c**) Heatmap showing gene expression differences between groups C and G. (**d**) Comparison of log2FC values for eight DEGs (*COL11A2*, *HOXC10*, *FN1*, *FBN1*, *AGT*, *FOS*, *SCD*, *SLC27A6*) between qPCR and mRNA-Seq results.

A total of nine yak longissimus dorsal muscle tissues from three groups were sequenced for transcriptomics; a total of 59.77 G of clean data was obtained. The effective data volume of each sample ranged from 5.87 G to 6.97 G, the distribution of Q30 bases ranged from 95.91 g to 96.81%, and the average GC content was 51.34%. This indicates that the data identification was accurate, the sequencing data quality was reliable, and it could be used for subsequent analysis. The results of PCA showed significant differences between groups G, Z, and C, as shown in Figure 3b, indicating that elevating the energy level of the diet significantly affected the yak longissimus dorsal muscle tissues.

The differentially expressed genes among the three groups were analyzed. A total of 570 DEGs were identified in ZvsC, among which 237 were upregulated and 333 were downregulated (Figure 3a). The significantly enriched items in the differentially expressed genes were identified through GO enrichment analysis. A total of 1965 GO items were significantly enriched in the differentially expressed genes of ZvsC. Among them, the ones mainly involved in fat or IMF regulation included protein homodimerization activity (*DCN*, *GADD45A*, *NR4A2*, and *FBLN5*), cell adhesion (*FN1*, *PCDH18*, *PRKCA*, *LAMC1* and *LAMA2*), positive regulation of cell migration (*PTK2B*, *PDGFRA*, *CSF1*, *F2R* and *CCAR1*), and extracellular space (*FABP3*, *FBLN5*, *LEP*, *RBP4*, and *Sfrp4*); KEGG enrichment analysis was conducted on the differentially expressed genes of ZvsC. The differentially expressed genes in the two comparison groups mainly enriched 284 KEGG pathways. Among them, those involving fat or IMF regulation mainly include glycerolipid metabolism (*DGAT2*, *PLPP3* and *LIPG*) and EGFR tyrosine kinase inhibitor resistance (*PDGFRA*, *VEGFA* and *IL6R*), PPAR signaling pathway (*PLIN2* and *FABP3*), Wnt signaling pathway (*Sfrp4*, *PRKCA* and *PPP3CA*), and PI3K-Akt signaling pathways (*FGFR1*, *PRKCA* and *TNC*).

A total of 226 DEGs were identified in GvsC, among which 75 were upregulated and 151 were downregulated (Figure 3c), significantly enriching 1147 GO entries. Among them, those involving fat or IMF regulation mainly included fextracellular space (*Sfrp4*). *LEP*, *FBN1*, and *LHB*, skeletal system development (*KLF10*, *GDF11*, *CDH11*, and HOXC10), and negative regulation of protein kinase activity (*FABP4*, *GADD45A*, and *TRIB1*). KEGG enrichment analysis was performed on the differentially expressed genes in the GvsC group, and 221 KEGG pathways were enriched. Among them, the ones involved in fat or IMF regulation mainly include the PPAR signaling pathway (*FABP4*, *PLIN1*, *SCD*, and *SLC27A6*), AMPK signaling pathway (*PFKFB3*), and Wnt signaling pathway (*Sfrp4*, *TP53* and *FBXW11*), as well as the AGE-RAGE signaling pathway in diabetic complications (*PLCD4*, *SERPINE1*, and *AGT*).

### 3.5. Validation of mRNA Sequencing Using RT-qPCR

As shown in Figure 3d, the expression of *COL11A2* in the LD of yaks in group Z and *HOXC10* gene in group G was up-regulated compared with that of yaks in group C, whereas the expression of *FN1*, *FBN1*, and *AGT* genes in the LD of group Z and the expression of *FOS*, *SCD*, and *SLC27A6* genes in group G was down-regulated. A total of eight DEGs in the LDs of yaks in groups C, Z, and G showed a consistent qPCR and mRNA-Seq data. Their expression patterns were consistent, indicating the reliability of the mRNA-Seq data in this study.

### 3.6. Correlation Analysis of Lipid Metabolomics and Transcriptomics

**Figure 4:**
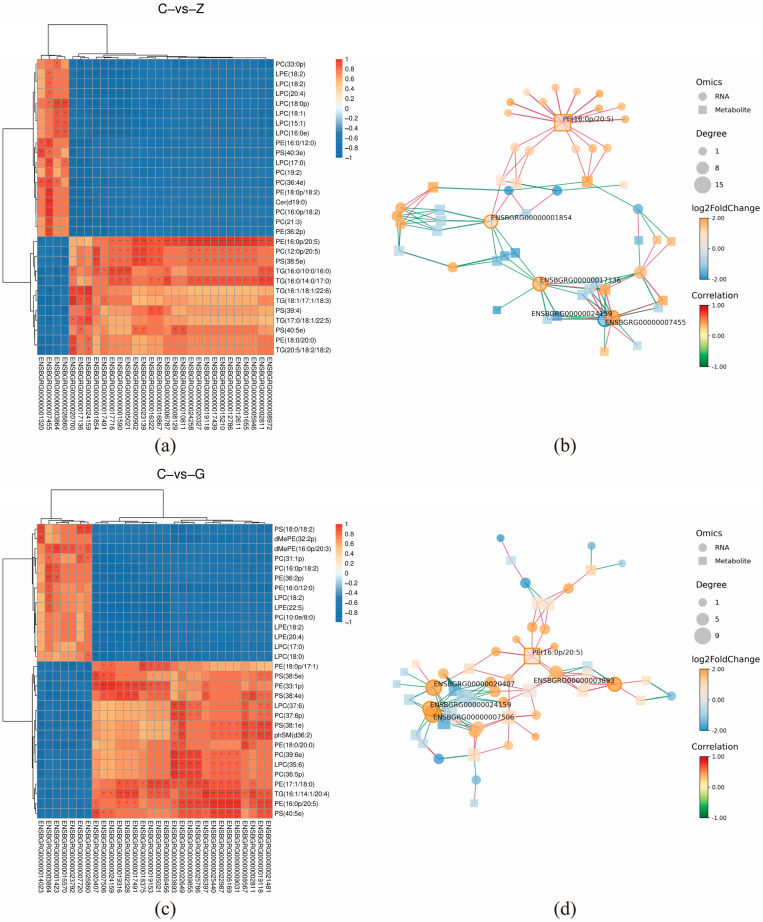
(**a**) Heatmap displaying the top 30 lipids and DEGs in yak dorsal muscle (groups C vs. Z). Red/blue indicate positive/negative correlations (“*” indicates that the correlation *p*-value is <0.05, “**” indicates that the correlation *p*-value is <0.01, and “***” indicates that the correlation *p*-value is <0.001.). (**b**) Network diagram of the top 20 lipids and differentially expressed genes in the longissimus dorsal muscle of yaks in group C vs. Z. Node shape, size, and color indicate the histological type, connectivity size, and corresponding degree of difference, respectively; and node color from red to blue corresponds to log2FoldChange from positive to negative. (**c**) Heatmap of the top 30 lipids and differentially expressed genes in the longissimus dorsal muscle of yaks from groups C and G. (**d**) Network diagram of the top 20 lipids and differentially expressed genes in the longissimus dorsal muscle of yaks in group C vs. G.

In order to uncover the regulatory mechanisms of intramuscular fat deposition in the LD of Pamir yaks, the first 30 differentially metabolized lipids and differential genes in the LD of the two comparison groups, ZvsC and GvsC, respectively, were correlated. The correlations between the differential genes and the lipid metabolites are shown in Figure 4; among them, key DEGs involved in the regulation of IMF content included *GADD45A*, *PDGFRA*, *RBP4*, *DGAT2*, *SFRP4*, and *FABP4* genes. The results showed significant positive correlation of *PDGFRA* and *RBP4* expression with PE (16:0p/20:5), PC (12:0p/20:5), and PS (38:5e), while *GADD45A* expression with PE (16:0p/20:5), TG (16:0/10:0/16:0), and TG (16:0/14:0/17:0) were significantly positively correlated with PE (16:0/14:0/17:0), where TG (16:0/14:0/17:0) was also significantly positively correlated with the expression of *PDGFRA*. In addition, the expression of *SFRP4* was significantly positively correlated with PE (16:0p/20:5), PC (12:0p/20:5), PS (38:5e), TG (16:0/10:0/16:0), while TG (16:0/14:0/17:0) were significantly positively correlated, and *DGAT2* expression was significantly positively correlated with TG (16:1/18:1/22:6), TG (18:/17:1/18:3), TG (17:0/18:1/22:5), and PS (40:5e). In the joint analysis of GvsC, *SFRP4* expression was significantly and positively correlated with PE (18:0p/17:1), PE (33:1p), PS (38:4e), PE (17:1/18:0), and TG (16:1/14:1/20:4), while *FABP4* expression was significantly and positively correlated with PE (33:1p), PS (38:4e), PE (17: 1/18:0), TG (16:1/14:1/20:4), and PE (16:0p/20:5) were significantly positively correlated.

### 3.7. Correlation of IMF, DEG and SDL

**Figure 5:**
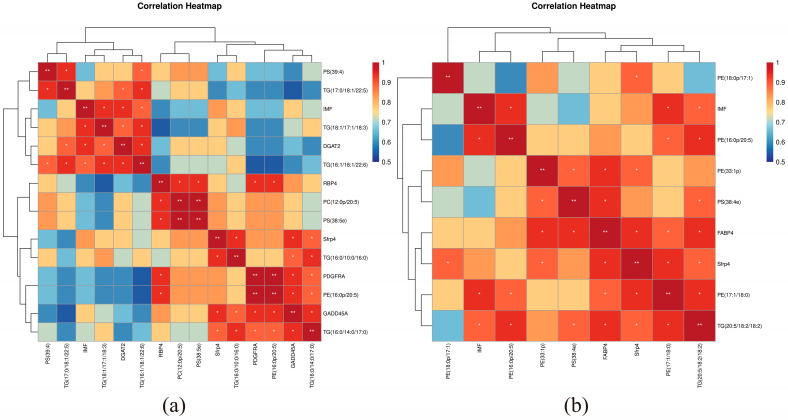
(**a**) Correlation heatmap of intramuscular fat content, DEGs and SDLs in yak dorsal muscle (groups C vs. Z). Red/blue indicate positive/negative correlations (“*” indicates that the correlation *p*-value is <0.05, “**” indicates that the correlation *p*-value is <0.01. (**b**) Corresponding analysis for groups C vs. G.

By analyzing the correlation of IMF with DEG and SDL, we revealed its regulatory mechanism on fat deposition in the LD of yak, and the results are shown in Figure 5a,b. In Z vs. C, TG (18:1/17:118:3), TG (16:1/18:1/22:6) and *DGAT2* showed significant positive correlation with IMF, and in G vs. C, PE (17:1/18:0), PE (16:0p/20:5), and TG (20:5/18:2/18:2) showed significant positive correlation with IMF.

## 4. Discussion

Yak meat has been popular among consumers for its rich nutrition. Yak meat is rich in protein and essential amino acids, but it is poorer than beef breeds in terms of eating quality, such as muscle tenderness, shear force, and cooking loss [32]. IMF content is closely related to shear force and tenderness, which is one of the determinants of meat characteristics [10]; therefore, it is important to excavate and elucidate the regulatory mechanism of IMF deposition.

In the present study, the IMF content of yak LD in group G exhibited a marked elevation compared to groups Z and C (*p* < 0.05), and its shear force displayed a substantial drop compared to groups Z and C (*p* < 0.05). In addition, the cooking loss of yak LD in groups C, Z, and G gradually decreased with the increase of energy level in the diet, and the diameter of muscle fibers of LD in group G was significantly lower than that in groups Z and C (*p* < 0.05). These results suggest that increasing the energy level in yak diets can effectively improve yak meat quality, especially in increasing IMF content. The results of this study are consistent with those of fattening cattle [33] and buffaloes [34], which both showed that IMF content increased with the increase of dietary energy.

Lipids are key factors in IMF deposition, and the lipids that promote IMF deposition mainly include phosphatidylserine (PS), triglycerides (TG), phosphatidylchloline (PC), and lysophosphatidylcholine (LPC) [35], of which TG is the main component of IMF. In the present study, the significant differential lipids in the two comparison groups were mainly TG, PS, and LPC, etc., and it was hypothesized that these significant differential lipids contributed to the differences in IMF deposition. Lipid deposition is mainly the synthesis of TG, etc., from esterified fatty acids of the glycerol skeleton, which is catalyzed by a range of enzymes and proteins, etc. [36]. Prior research has demonstrated a number of candidate genes (*PPAR*, *DAGT*, and *AGPAT*) associated with IMF deposition, and most of these genes have been linked to the deposition of lipids such as TG and GP [37], suggesting that these genes serve as a critical determinant of IMF deposition by regulating lipid metabolism.

Transcriptome analysis identified six key DEGs (*SFRP4*, *FABP4*, *GADD45A*, *PDGFRA*, *RBP4*, and *DGAT2*) that regulate IMF content. *SFRP4* acts as an antagonist of the Wnt/β-catenin signaling pathway [38,39] and promotes adipogenesis through the upregulation of adipogenic gene expression; its expression trend is consistent with IMF content, which serves as a critical determinant of IMF deposition [40], consistent with the positive correlation between IMF content and *SFRP4* in bovine LD reported by Tan et al. [41]. *FABP4* is a protein expressed in adipocytes, which plays an important role in fatty acid transport and lipid hydrolysis [42], and a strong positive relationship was observed between the expression level and meat quality texture traits, IMF deposition, and adipocytogenesis [43,44,45]. It was also found that lncFABP4, which is transcribed from the antisense strand of *FABP4*, is upregulated in the lipogenic differentiation of intramuscular preadipocytes and thereby promotes preadipocyte differentiation by regulating *FABP4* expression [46]. Notably, both *SFRP4* and *FABP4* were significantly positively correlated with PE (17:1/18:0) and TG (20:5/18:2/18:2), which were also highly correlated with IMF content, suggesting that these genes may synergistically promote IMF deposition by regulating specific lipid metabolism.

*GADD45A* was differentially expressed in the LD of two different pig breeds [45], and Pearson correlation analysis showed that the level of *GADD45A* expression increased with increasing IMF content [47]. In Berkshire pigs, the SNP for *GADD45A* was significantly correlated with IMF [48], and its expression revealed a statistically significant positive correlation with genes related to lipidogenic differentiation (*FABP4*, *CEBPA*, and *CEBPB*) [49]. The expression level of *GADD45A* was higher in breeds with high IMF deposition compared to lean breeds with low IMF deposition [50]. In addition, the present study found that *GADD45A* showed strong positive correlation with the *PDGFRA* gene and lipid TG (16:0/10:0/16:0), PE (16:0p/20:5), and TG (20:5/18:2/18:2), suggesting that *GADD45A* is a potentially important gene in regulating IMF deposition.

*PDGFRA*, an important biomarker of preadipocytes, was expressed at significantly higher levels in adipose pig LD than in lean pig and mediated adipogenesis through the Erk signaling pathway [51]; furthermore, fattened Angus cattle contained significantly higher levels of *PDGFRA* than Nellore cattle [52], confirming that *PDGFRA* is a beef intramuscular adipocyte progenitor cell marker [53]. In the context of this investigation, *PDGFRA* was significantly and positively correlated with *GADD45A*, *RBP4*, PE (16:0p/20:5), and TG (16:0/14:0/17:0), suggesting that *PDGFRA* may modulate TG metabolic pathways through synergistic interactions with *GADD45A* and RBP4, which may in turn promote IMF deposition.

*RBP4* is an adipokine and fatty acid transport protein that plays an important role in fat deposition. Studies have shown that plasma levels of *RBP4* in 8-month-old cattle were significantly higher than those in 18-month-old cattle, suggesting that *RBP4* is involved in adipose tissue development [54]. In addition, *RBP4* has been identified as one of the key genes involved in lipid metabolism in intramuscular adipocytes in the Piedmontese × Hereford and Wagyu × Hereford crosses and was positively associated with IMF deposition in Brahman cattle [55]. Another study showed that *RBP4* was differentially expressed in LD of cattle before and after 3 months of fattening [56]. In the context of this investigation, *RBP4* was found to be significantly and positively correlated with lipids such as PC, PS, and PE, further confirming the important role of *RBP4* in lipid metabolism and IMF deposition in cattle.

*DGAT2*, as the final step in catalyzing triglyceride synthesis [57], showed a strong positive correlation between its expression level and IMF content [58,59,60]. *DGAT2* gene polymorphism was found to affect the tenderness of yak meat and could be used as a genetic marker to improve the tenderness of yak meat [61]. Cellular study level confirmed that *DGAT2* overexpression significantly increased TAG deposition in intramuscular precursor adipocytes of goats, while silencing expression of *DGAT2* decreased TAG content [62]. This result was validated in 3T3-L1 adipocytes [63], suggesting that *DGAT2* plays a key role in IMF deposition. The present experiment showed that *DGAT2* not only presented a statistically significant positive correlation with IMF, but also a significant positive correlation with TG (18:1/17:1/18:3), further confirming that *DGAT2* is a key candidate gene for regulating IMF deposition.

## 5. Conclusions

This study mainly discussed the regulation of the energy level in the diet on the deposition of IMF in yak LD. The results show that diets with higher energy levels can improve meat quality to a certain extent and provide consumers with higher quality yak meat products. This study screened some differential genes and lipid metabolites. Among them, the IMF and differential lipids of yak meat were mainly regulated by the *SFRP4*, *FABP4*, *GADD45A*, *PDGFRA*, *RBP4*, and *DGAT2* genes, which can be used as a theoretical basis to reveal the regulatory effect of IMF deposition in yak meat and improve the deposition of IMF in yak meat.

## Figures and Tables

**Table 1:** Pamir yak fattening concentrate feed formula table (DM).

Item	Low Energy Supply (C)	Medium Energy Supply (Z)	High Energy Supply (G)
Ingredient (%)			
Corn	37.4	59.5	68.8
46 Cotton meal	0	9.2	10
43 Soybean meal	0	12.1	13
Soybean Oil	0	0	1.2
Bran (especially wheat)	7.6	11.7	2
Syrup	0	2.5	0
Premix ②	5	5	5
Total	100.00	100.00	100.00
Nutrient Level ①			
Crude protein (%)	9.95	19.38	19.17
Net energy of weight Gain (MJ/Kg)	0.75	1.38	1.53

Note: ① Crude protein level is measured value (Kjeldahl method), and net weight gain energy is theoretical calculated value. ② Each kilogram of premix contains Fe 2500 mg, Zn 1000 mg, Cu 1000 mg, Mn 1000 mg, Se 7.5 mg, I 20 mg, Vitamin A 300,000 IU, Vitamin D 5000 IU, Vitamin E 4000 IU.

**Table 2:** Primer sequences.

Gene	Primer Sequences (5′–3′)	Annealing Temperature (°C)
*COL11A2*	F: ACCACCAAGACTTCACAGGC R: GGGGTGGGGTCCTGATAAT	60.3
*HOXC10*	F: CGACAACGAAGCAAAAGAGGAG R: TCCAGCGTCTGGTGTTTAGT	60.3
*FN1*	F: CCCTGGTGTCACAGAAGCTA R: CTGGGGGAGCTCATCTGTCTT	60.3
*FBN1*	F: TGGCTCCAGATCCATCCAACA R: CCTTTCTGGCACAGACAGTGA	60.3
*AGT*	F: GACCCAAATCTCGCTGCTGA R: GAAGCCCCTCATCTTTCCTTGG	60.3
*FOS*	F: AGGGGCAAGGTAGAACAGTTG R: CTAGTTGGTCTGTCTCCGCTT	60.3
*SCD*	F: TCCCGACGTGGCTTTTTCTT R: CACCAGGTTTGTAGTACCTCCT	60.3
*SLC27A6*	F: TGTGGTTGTGCCAGGTTATGA R: AAAACTGTGGACGAGCGTAAG	60.3
*β-actin*	F: GCAGGTCATCACCATCGG R: CCGTGTTGGCGTAGAGGT	60.3

**Table 3:** Determination of meat quality traits in yaks fed diets with different energy levels.

Item	Group C	Group Z	Group G
pH*_45min_	6.99 ± 0.00 ^a^	6.7 ± 0.02 ^b^	5.49 ± 0.03 ^c^
L*_45min_	9.25 ± 0.03 ^c^	9.48 ± 0.02 ^a^	9.32 ± 0.04 ^b^
a*_45min_	20.78 ± 0.03 ^c^	21.31 ± 0.22 ^b^	21.70 ± 0.11 ^a^
b*_45min_	3.39 ± 0.03 ^c^	3.54 ± 0.02 ^b^	3.82 ± 0.03 ^a^
pH*_24h_	6.42 ± 0.02 ^a^	5.64 ± 0.03 ^b^	5.28 ± 0.02 ^c^
L*_24h_	9.78 ± 0.06 ^b^	10.75 ± 0.10 ^a^	10.79 ± 0.05 ^a^
a*_24h_	21.39 ± 0.13 ^b^	21.51 ± 0.07 ^b^	23.49 ± 0.02 ^a^
b*_24h_	4.50 ± 0.12 ^b^	3.95 ± 0.06 ^c^	4.77 ± 0.00 ^a^
Steaming loss (%)	30.42 ± 1.15	29.71 ± 2.98	27.67 ± 2.86
Shearing force (kg f)	100.90 ± 0.61 ^a^	98.27 ± 1.78 ^a^	83.69 ± 1.89 ^b^
Intramuscular fat (g/100 g)	0.97 ± 0.02 ^b^	1.25 ± 0.07 ^b^	2.04 ± 0.42 ^a^

Note: Different lowercase letters marked in the table indicate significant differences (*p* < 0.05).

## Data Availability

Data used in this study are available from the corresponding author on reasonable request.

## Abbreviations

The following abbreviations are used in this manuscript:

IMF	Intramuscular fat
LD	Longissimus dorsi muscle
DEG	Differentially expressed genes
SDL	Significant differential lipids
QC	Quality control
PCA	Principal component analysis
OPLS-DA	Orthogonal partial least squares discriminant analysis
KEGG	Kyoto Encyclopedia of Genes and Genomes
